# Asymmetric and non-uniform evolution of recently duplicated human genes

**DOI:** 10.1186/1745-6150-5-54

**Published:** 2010-09-08

**Authors:** Alexander Y Panchin, Mikhail S Gelfand, Vasily E Ramensky, Irena I Artamonova

**Affiliations:** 1 M.V. Lomonosov Moscow State University, Faculty of Bioengineering and Bioinformatics, Vorobyevy Gory 1-73, Moscow, 119992, Russia; 2 A.A. Kharkevich Institute for Information Transmission Problems, Russian Academy of Science, Bolshoi Karetny 19, Moscow, 127994, Russia; 3 V.A. Engelhardt Institute of Molecular Biology, Russian Academy of Sciences, Vavilova 32, Moscow, 119991, Russia; 4 N.I. Vavilov Institute of General Genetics, Russian Academy of Science, Gubkina 3, Moscow, 119991, Russia

## Abstract

**Background:**

Gene duplications are a source of new genes and protein functions. The innovative role of duplication events makes families of paralogous genes an interesting target for studies in evolutionary biology. Here we study global trends in the evolution of human genes that resulted from recent duplications.

**Results:**

The pressure of negative selection is weaker during a short time immediately after a duplication event. Roughly one fifth of genes in paralogous gene families are evolving asymmetrically: one of the proteins encoded by two closest paralogs accumulates amino acid substitutions significantly faster than its partner. This asymmetry cannot be explained by differences in gene expression levels. In asymmetric gene pairs the number of deleterious mutations is increased in one copy, while decreased in the other copy as compared to genes constituting non-asymmetrically evolving pairs. The asymmetry in the rate of synonymous substitutions is much weaker and not significant.

**Conclusions:**

The increase of negative selection pressure over time after a duplication event seems to be a major trend in the evolution of human paralogous gene families. The observed asymmetry in the evolution of paralogous genes shows that in many cases one of two gene copies remains practically unchanged, while the other accumulates functional mutations. This supports the hypothesis that slowly evolving gene copies preserve their original functions, while fast evolving copies obtain new specificities or functions.

**Reviewers:**

This article was reviewed by Dr. Igor Rogozin (nominated by Dr. Arcady Mushegian), Dr. Fyodor Kondrashov, and Dr. Sergei Maslov.

## Background

Gene duplications and subsequent differentiation of duplicated genes are important processes in the evolution of living organisms [1]. Gene duplication promotes phenotypic diversity and is involved in the manifestation of many genetic disorders [2]. Evolutionary histories of multigene families have been studied in a variety of organisms on the scale of individual families [3-7] as well as in genome-wide analyses [8-12].

Regarding gene duplication and evolution, one of the best studied organisms is the budding yeast *S. cerevisiae*. This organism experienced a recent whole-genome duplication event [13] that, together with smaller segment duplications provided material for comparison of duplicated genes of different origin. The results of these studies are somewhat contradictory. Paralogous genes originating from whole-genome duplications have weaker phenotypic effects when deleted and are less divergent in function [14]. The whole genome duplication event in the *Saccharomyces *lineage was followed by a significantly increased rate of the evolution of duplicated genes [15]. In several studies, this evolution was shown to be significantly asymmetric for different genes copies: one copy evolved faster than the other [15-17]. Genes duplicated in yeast evolve slower on the sequence level in other species [10]. The probability to cause lethality is higher for *S. cerevisiae *singleton genes if they have no orthologs duplicated in any of the other yeast species [11].

Cross-species analysis of duplicated genes demonstrated that the dN/dS ratio is higher in singleton genes, compared to the dN/dS ratio for genes with two or more copies per genome [18] (here dN is the number of non-synonymous substitutions per non-synonymous site, dS is the number of synonymous substitutions per synonymous site; smaller dN/dS values indicate stronger negative selection). Strong negative selection on genes with multiple copies could result from the initial importance of these genes. On the other hand, pairs of duplicated genes show weaker negative selection than pairs of orthologous genes that have separated at the same time [19]. One more cross-species study reports that most duplicated genes experience a short post-duplication period of relaxed neutral selection [12]. While most of them become pseudogenes, the remaining duplicates start experiencing strong purifying selection. It has been proposed that neofunctionalization is a common fate for duplicates that have been created by a single duplication event and avoided pseudougenization, in contrast to members of large gene families [20].

The existing publications concerning the asymmetry of the evolution of human paralogs are also somewhat controversial. Whereas no significant evidence of asymmetry was found in a multispecies comparative study dealing with organisms from all three domains of life [19], a significant level of asymmetry in the evolutionary rates in pairs of human paralogous genes was observed in a study where pairs of recent paralogs were compared with outgroup genes from the mouse genome [21]. Such incompatible findings could be due to differences in the methods used. Asymmetry was found in the evolution of rodent duplicated genes demonstrating that a gene copy duplicated to a new genomic location tend to evolve faster [22]. A study on the frog *X. laevis *demonstrated that genes from paralogous pairs are usually expressed in similar locations and developmental timings, but at significantly different levels [23].

Here we describe major trends in the evolution of recently duplicated human genes. Instead of using genes from other mammalian species as outgroups, as done in most studies, we focus on human gene families containing three or more genes. Hence, relatively distant family members can be used as outgroups for genes that have undergone recent duplications. This approach allows us to avoid problems with precise identification of orthology relations and to calculate individual evolutionary rates of genes evolving in the same genome, i.e., on the same evolutionary distances and under the same conditions. We show that there is a global trend in the decrease of dN/dS values with growing dS values, both for the majority of individual genes and for gene families. We also report that 18% of recent human paralogous pairs evolve with significant asymmetry and that faster evolving gene copies accumulate functional mutations at a significantly higher proportion then slowly evolving genes from the same pairs or genes from non-asymmetrically evolving pairs. The observed asymmetry is unlikely to be caused by pseudogenisation, but more likely to be a result of functional divergence of genes.

## Results

### Estimation of individual dN and dS rates

**Figure 1:**
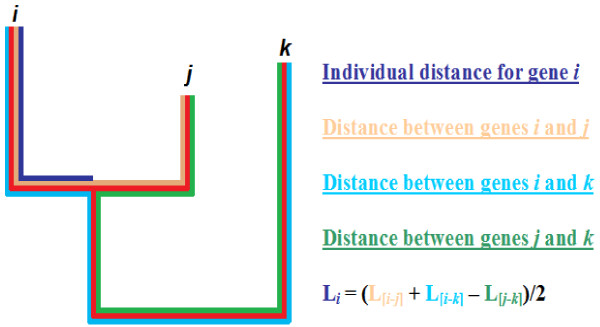
**Relationships between gene i, its sister j and cousin k**. L_i _= (L_[i-j] _+ L_[i-k] _- L_[j-k]_)/2, where L_i_ is the individual distance (T, dS or dN) for gene i. L_[i-j] _is the distance between gene i and its sister j. L_[i-k] _is the distance between gene i and its cousin k. L_[j-k] _is the distance between the sister and the cousin of gene i.

To calculate individual, gene-specific, dN and dS rates we have selected the closest paralog for each gene within its family. We refer to this gene as the "sister" of the initial gene. For such pair of genes we find the closest outgroup within the same family. We refer to this outgroup as the "cousin" gene. By definition, the cousin gene is positioned outside the branch containing the sister gene pair in the phylogenetic tree, *i.e*., is an outgroup (Figure 1).

More formally, the dS value produced by the PAML codeml program was used to determine the closest neighbors of each gene. This value represents the evolutionary time since the separation of two sequences. In addition to the identity restrictions (see Methods), only genes with 0.01 ≤ dS ≤ 3 were compared, to ensure that very close and very distant sequences are not paired in the analyzed set. If the closest neighbor was too close (dS < 0.01) to the given gene, the next, more distant, neighbor was considered as the sister gene. The cousin was determined as follows (Figure 1). Let gene *j *be the sister of a given gene *i*. Then gene *k *is the cousin of gene *i*, if dS_[*i*-*k*] _> dS_[*i*-*j*] _and dS_[*j*-*k*] _> dS_[*i*-*j*]_, where dS_[*i*-*k*]_, dS_[*i*-*j*]_, and dS_[*j*-*k*] _are the values of dS between genes *i *and *k*, *i *and *j*, *j *and *k*, respectively. These criteria ensure that gene *k *is placed outside the branch containing genes *i *and *j*, thus gene *k *is a valid outgroup for the pair of genes *i *and *j *(Figure 1). If a gene pair did not have an appropriate cousin, it was not considered further.

For each gene from a pair with a cousin we calculated individual dN (dN_I_) and dS (dS_I_) values using the following formulas (illustrated in Figure 1):

$$\begin{array}{l} {\text{dN}}_{\text{I}}=\left({\text{dN}}_{\left[i-k\right]}+{\text{dN}}_{\left[i-j\right]}-{\text{dN}}_{\left[j-k\right]}\right)/\text{2};\\ {\text{dS}}_{\text{I}}=\;\left({\text{dS}}_{\left[i-k\right]}+{\text{dS}}_{\left[i-j\right]}-{\text{dS}}_{\left[j-k\right]}\right)/\text{2}, \end{array}$$

where, as above, the indices [*i*-*j*], [*i*-*k*], [*j*-*k*] stand for the pairwise values of dN or dS for the gene *i*, its sister *j *and its cousin *k*. The individual dN_I _and dS_I _values therefore represent the numbers of synonymous or non-synonymous substitutions per site that occurred from the moment of the last duplication event for the current gene.

In this study the individual evolutionary rates for each gene were used instead of pairwise dN and dS values commonly used in the literature. The outgroup information was taken into account in the calculations of individual dN and dS values to increase their accuracy. This also allowed us to compare the dN and dS values of individual genes, rather than gene pairs within a gene family. A similar method was used in a study of yeast *Saccharomyces cerevisiae *duplicated genes by Kim and Yi [24], but sequences of orthologous genes were used instead of paralogous genes as outgroups. Here we focus on genes that have evolved after recent duplications and, therefore, have relatively small dS values: only genes with individual dS values between 0.005 and 0.6 were used for the individual dN and dS analyses.

### Individual dN and dS values of paralogous genes

We identified and evaluated 97 gene families that matched the criteria described in the Methods section. The families contained 511 genes, 446 of which had both a sister and a cousin. For these 446 genes, individual dN and dS were calculated. The average individual dS value was 0.18 and the average individual dN value was 0.05. Single-exon genes represented 59% of genes for which individual dN and dS were calculated, that is roughly the same fraction as in the complete dataset (56.5%) (see Methods). The coding sequence alignments of paralogous gene families had the average length of ~1100 nucleotides (ranging from 153 to 5775 nucleotides). The smallest observed substitution rate between a gene and its sister was 13 substitutions in 1263 nucleotides (1%) and the highest substitution rate was 332 substitutions in 897 nucleotides (37%), the average being 160 substitutions in 1114 nucleotides (14%).

GOstat [25] comparison of all genes from the constructed families (511 in total) with the human transcriptome revealed overrespresentation of olfactory receptors, genes involved in the ectoderm and nervous system development, signal transduction, cell adhesion and defense response in our dataset (P < 10^-5^). Genes involved in metabolic processes, nucleotide binding, biological regulation and protein modification were underrepresented (P < 10^-5^).

**Figure 2:**
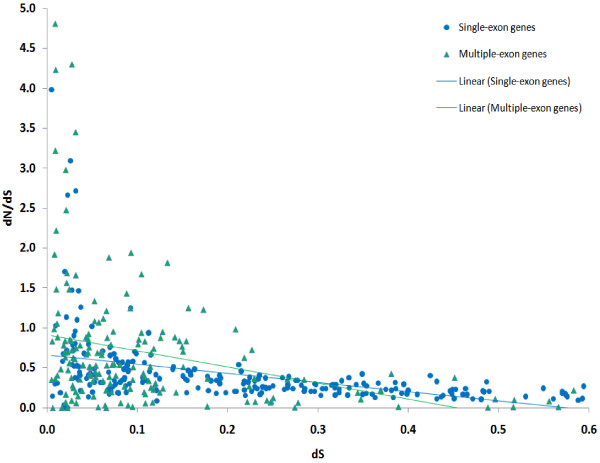
**Individual dS and dN/dS of genes**. Single-exon genes are shown as blue circles, genes with multiple-exons are represented by green triangles. Only genes with individual 0.005 ≤ dS ≤ 0.6 are shown. Linear regression trend lines are provided for single-exon and multiple-exon genes separately (regression coefficients not shown).

**Figure 3:**
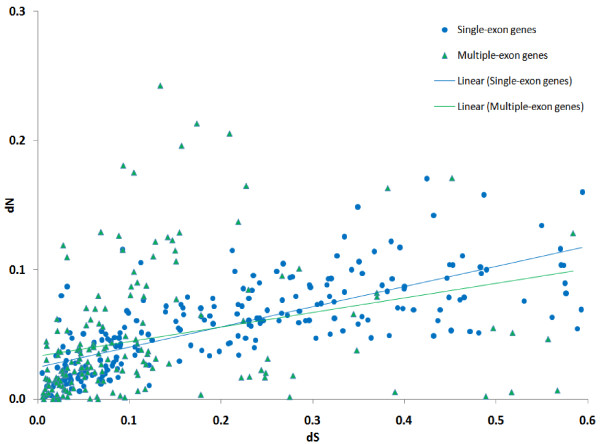
**Individual dS and individual dN of genes**. Single-exon genes are shown as blue circles, genes with multiple-exons are represented by green triangles. Only genes with individual 0.005 ≤ dS ≤ 0.6 are shown. Linear regression trend lines are provided for single-exon and multiple-exon genes separately (regression coefficients not shown).

The dependency between dN/dS (strength of selection) and dS (approximating the time from the last duplication event) for individual genes is shown in Figure 2. The dependency between individual dN and dS is shown in Figure 3. As mentioned above, only genes with 0.005 ≤ dS ≤ 0.6 are shown (405 genes). There is an obvious trend showing that dN increases slower for larger values of dS (the linear regression coefficient for dN/dS and dS is -1.37; 95% confidence interval is (-2.43; -0.30)). This is true both for the subset of single-exon genes and for genes with multiple exons. Note that, due to the method's limitations, individual dS estimates are more reliable for genes with lower dS. However, even if we consider the interval dS ≤ 0.2, where the method is most reliable, the tendency of dN/dS decrease as dS increases persists.

**Figure 4:**
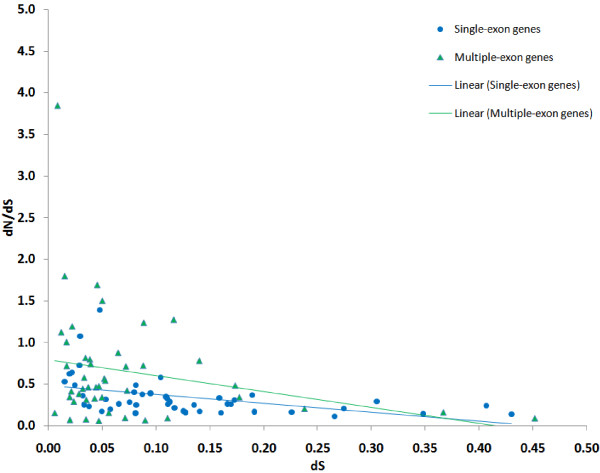
**The average dN/dS for gene families and their average dS**. Single-exon genes are shown as blue circles, genes with multiple-exons are represented by green triangles. Only genes with individual 0.005 ≤ dS ≤ 0.6 are shown. Linear regression trend lines are provided for single-exon and multiple-exon genes separately (regression coefficients not shown).

**Figure 5:**
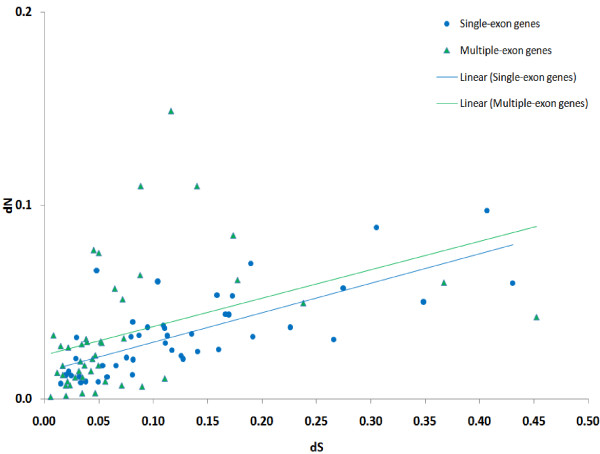
**The average dN for gene families and their average dS**. Single-exon genes are shown as blue circles, genes with multiple-exons are represented by green triangles. Only genes with individual 0.005 ≤ dS ≤ 0.6 are shown. Linear regression trend lines are provided for single-exon and multiple-exon genes separately (regression coefficients not shown).

Similar trends can be found for the average values of dN, dS and dN/dS for paralogous gene families (Figure 4 and Figure 5). If we use dN/dS as an estimate of selective pressure and dS as an estimate of time, we can state that older paralogs are evolving under stronger negative selection, while younger paralogs often evolve under weaker negative selection, and sometimes even with dN/dS > 1. On average, young gene families undergo less pressure of negative selection than families mostly consisting of genes with larger individual dS_I_.

We also calculated individual dN and dS using branch-specific values of dN and dS calculated by codeml (model = 2; 2 or more dN/dS values for branches; trees created by clustalw). We obtained plots similar to Figure 2 and Figure 3. The linear regression coefficient for dN/dS and dS was -1.18; 95% confidence interval (-2.05; -0.32).

As an additional control we restricted the sample to genes that had at least 100 substitutions, when compared to their sister gene. This was done to ensure that the observed trends are not caused by discrete substitution counts. The linear regression coefficient for dN/dS from dS in this subsample of 232 genes remained negative and was equal to -1.31; 95% confidence interval (-1.94; -0.69).

### Asymmetry at non-synonymous sites

144 gene pairs had a valid cousin (see Methods). We calculated the number of amino acid substitutions in the encoded proteins, using the cousin as an outgroup. Twenty six gene pairs of genes from 144 (18%) were found to be significantly asymmetric by the number of amino acid substitutions in the encoded proteins (P < 0.05). The P-value of the hypothesis to have 26 or more gene pairs out of 144 being asymmetric only by chance, assuming that the probability for a gene pair to be asymmetric is p = 0.05, as defined in Methods, is 1.33 × 10^-8^. This shows that the asymmetry is a pronounced trend in the evolution of duplicated genes: members of almost one fifth of gene pairs evolve at significantly different rates. These 26 gene pairs are listed in Table 1. Single-exon genes constitute 54% of genes from the asymmetrically evolving pairs. This is very close to the average fraction of single-exon genes in considered 144 pairs (58%).

**Table 1:** Asymmetrically evolving gene pairs.

Copy evolving fast	mRNA	EST	Mutations	Copy evolving slow	mRNA	EST	Mutations	Function
[RefSeq: NM_182537]	7	0	52	[RefSeq: NM_182589]	10	0	8	Serotonin receptor

[RefSeq: NM_001083538]	3	4	29	[RefSeq: NM_001101]	230	26018	1	Actin beta

[RefSeq: NM_002030]	9	76	51	[RefSeq: NM_001005738]	12	146	15	Formyl peptide receptor

[RefSeq: NM_001009562]	16	38	77	[RefSeq: NM_019844]	12	51	34	Organic anion transporter

[RefSeq: NM_001001659]	2	0	32	[RefSeq: NM_012365]	3	0	12	Olfactory receptor

[RefSeq: NM_000896]	12	94	36	[RefSeq: NM_001082]	11	70	15	Cytochrome P450

[RefSeq: NM_152631]	3	24	64	[RefSeq: NM_001013736]	2	22	36	Unknown

[RefSeq: NM_001004692]	1	0	13	[RefSeq: NM_001004695]	3	0	2	Olfactory receptor

[RefSeq: NM_145660]	17	90	46	[RefSeq: NM_145640]	18	627	24	Apolipoprotein

[RefSeq: NM_001006938]	2	22	10	[RefSeq: NM_001006933]	6	81	1	Transcription elongation factor

[RefSeq: NM_032098]	157	856	48	[RefSeq: NM_032099]	157	856	27	Protocadherin gamma

[RefSeq: NM_001004737]	3	0	11	[RefSeq: NM_001004736]	4	0	2	Olfactory receptor

[RefSeq: NM_001004743]	1	0	37	[RefSeq: NM_001005282]	3	0	20	Olfactory receptor

[RefSeq: NM_005557]	9	464	40	[RefSeq: NM_000526]	16	1853	8	Keratin 16

[RefSeq: NM_020040]	2	1	24	[RefSeq: NM_177987]	19	2	3	Tubulin, beta polypeptide

[RefSeq: NM_002699]	4	20	15	[RefSeq: NM_006236]	1	2	0	POU class 3 homeobox

[RefSeq: NM_017534]	11	451	64	[RefSeq: NM_005963]	6	205	27	Myosin, heavy chain

[RefSeq: NM_001004482]	3	0	19	[RefSeq: NM_001004481]	3	0	2	Olfactory receptor

[RefSeq: NM_001004454]	2	0	50	[RefSeq: NM_001005236]	1	0	20	Olfactory receptor

[RefSeq: NM_001013435]	5	26	12	[RefSeq: NM_001015038]	2	16	0	P antigen family

[RefSeq: NM_000150]	15	59	26	[RefSeq: NM_002034]	4	1	6	Fucosyltransferase

[RefSeq: NM_001005479]	3	0	27	[RefSeq: NM_001005514]	1	0	7	Olfactory receptor

[RefSeq: NM_001005519]	2	0	31	[RefSeq: NM_054105]	2	0	11	Olfactory receptor

[RefSeq: NM_001005471]	1	0	34	[RefSeq: NM_030904]	1	0	15	Olfactory receptor

[RefSeq: NM_012373]	3	0	17	[RefSeq: NM_002551]	2	3	5	Olfactory receptor

[RefSeq: NM_032089]	157	856	74	[RefSeq: NM_014004]	157	856	49	Protocadherin gamma

The gene pairs asymmetric by the number of non-synonymous substitutions are shown. The number of substitutions is only for non-synonymous substitutions that were acquired in this gene, when compared to its sister and cousin gene (see Methods). Data for mRNA and EST count is presented according to the UniGene database.

In six out of the 26 asymmetric gene pairs, the faster evolving copy had individual dN/dS > 1, not observed among the slower evolving copies. Although rapid divergence of gene expression profiles for paralogs had been reported before [26], we did not find a significant difference in the number of ESTs or mRNAs present in the UniGene database [27] between the slowly and rapidly evolving copies from the 26 asymmetric gene pairs. According to a GOstat comparison, genes from asymmetrically evolving gene pairs are not significantly different (P < 0.01) from other genes of the initial dataset in terms of protein function.

### Asymmetry at synonymous sites

We identified nine pairs of paralogous genes asymmetric according to the number of synonymous substitutions (P < 0.05). The P-value of the hypothesis to have 9 or more gene pairs out of 144 being asymmetric only by chance, assuming that the probability for a gene pair to be asymmetric is p = 0.05, is 0.295. Therefore, we cannot reject the hypothesis that this observation is due to chance. Four gene pairs are asymmetric by the number of substitutions at both synonymous and non-synonymous sites.

### PolyPhen analysis

Computational approaches have been shown to be able to predict amino acid substitutions in proteins that are functional and therefore are subject to purifying selective pressure [28,29]. We used a new version of the PolyPhen tool [30] for the analysis aimed at quantification of the fraction of potentially functional non-synonymous substitutions in various classes of paralogous genes. In 288 proteins encoded by the genes that were used in the asymmetry study, 8104 directed amino acid substitutions were classified by PolyPhen into "damaging" and "neutral" ones. Out of 8104 new variants, 542 (6.7%) were predicted to be damaging with 128 of them in asymmetric gene pairs. In turn, 127 of 128 damaging variants were present in the faster evolving gene copy and only one in the slower evolving copy. The number of damaging variants is overrepresented in the faster evolving copies of asymmetric genes (p < 0.001) and underrepresented in the slower evolving copies (p < 0.001) compared to the set of non-asymmetrically evolving gene pairs.

### Pride analysis

One gene from each asymmetrically evolving pair acquires mutations at a very high rate. This is suspicious because this could imply that some of these genes are recent pseudogenes. To address this possibility, we analyzed additional evidence that faster evolving genes from asymmetric pairs are functional. In particular, we curated all corresponding mRNAs and ESTs and confirmed sufficient experimental evidence of transcription for all these genes. Additionally, we tried to confirm the existence of gene products on the protein level using Pride [31,32], a database that contains experimental reads of protein fragments. We established that 23 out of 26 faster evolving copies of genes from asymmetric gene pairs are validated by unique amino acid sequences in Pride. For the remaining three genes, neither their nor their sister's amino acid sequences were observed. Overall, this confirms that the observed asymmetry is not due to pseudogenization.

## Discussion

A number of publications demonstrated that certain synonymous sites are evolving under the pressure of selection. This selection could be a result of differences in tRNA concentrations [33], translation kinetics [34], translation accuracy [35,36], and protein folding [37] that are dependent on codon usage, as well as the existence of conserved exonic splicing enhancers and silencers [38,39], or some other yet unknown factors. However, we found no significant evidence for asymmetry in the distribution of synonymous substitutions within pairs of paralogs. Synonymous substitutions are distributed much more evenly among genes than substitutions leading to amino acid change.

While it is possible that some synonymous sites experience selection, it is much weaker than the selection at non-synonymous sites for genes evolving after recent duplication. On average, individual dS can be considered as an estimate of the time passed since the last duplication event, while the value of dN/dS reflects the strength of natural selection.

As shown in Figure 3, most genes from paralogous gene families are subject to negative selection, but the strength of this selection is substantially weaker if the latest duplication event was recent. This observation holds both for single-exon and multiple-exon genes. This is correct for gene families as well: younger families tend to evolve under weaker negative selection. Thus, duplicated genes change mainly during their early evolution. These findings are in agreement with the speculative scenario described below.

Immediately after duplication, the pressure of negative selection weakens and pairs of genes start evolving rapidly, possibly until both acquire important, but different, physiological roles. At this point strong negative selection is restored and the genes acquire less non-synonymous substitutions. Duplicated genes that are not beneficial are likely to be lost during their early (independent) evolution and mechanisms, as the one described above, could be a possible way for the fixation of beneficial duplications. Our results are well in line with other studies; in particular, similar trends have been observed by Koonin et al. [18], with orthologs from mice used as outgroup genes.

However, this evolutionary scenario, while the most common, is not the only possible one. We also observed strong evidence for the asymmetry in the distribution of amino acid substitutions between proteins encoded by pairs of closest paralogs. 18% of such gene pairs are significantly asymmetric. This reveals the second pronounced trend in the evolution of paralogous genes: after a duplication event, one gene remains practically unchanged, whereas the other one evolves quickly. We can speculate that these could be the cases when the unchanged copy of a gene preserves the initial function, while the other one evolves to obtain a different function. The PolyPhen analysis provides evidence that the observed asymmetry is caused by an increased number of new potentially functional variants in one copy of a gene and the decrease of the frequency of such substitutions in the other copy. We believe that this is an evidence of a radical change of function in most cases, rather than pseudogenization. Indeed, all genes from the asymmetrically evolving gene pairs are transcribed (have validated mRNAs and, usually, ESTs) and have no premature stop codons. Furthermore, most of them have experimentally identified amino acid products. We were not able to find any evidence of pseudogenization for these genes based on available data.

Some observations of the asymmetry in the evolution of human genes have been reported before, for example in [21] where orthologous genes from the mouse genome were used as outgroups. One of the novelties of our study is using paralogous genes instead of orthlogs as outgroups. We believe this to be more correct, because in this case all three compared genes evolve in the same organism, and therefore in the same conditions and environment. Also, this usually allows us to use a closer partner for comparison: in over 60% of the triplets studied, the human paralog was closer to a gene and its sister than the closest ortholog from mouse (by protein sequence identity). Besides, in this case one does not encounter the problem of precise resolution of orthology relationships. Yet, there are drawbacks. The requirement for gene families with three or more genes in each family decreases the sample size and introduces a bias towards families with a large number of genes. The presence of a third paralog might also affect the pattern of substitutions between other duplicated genes, but this is a problem for most studies on duplicated genes. One could exclude families with more than two paralogs but this would create a bias in the other direction and further reduce the sample size.

We did not observe any correlation between the mutation rates of asymmetrically evolving genes and gene expression level differences. We found no evidence that mRNA abundance significantly influences the evolution rate of paralogous genes.

## Conclusions

There seems to be a major trend in the evolution of human paralogous genes and gene families: negative selection pressure increases over time after a duplication event. This trend is not biased by small substitution counts. Significant asymmetry was observed in the evolution of 18% pairs of human paralogs when a third paralog is used as an outgroup. This shows that in many cases one of two gene copies remains practically unchanged, while the other copy evolves quickly, acquiring a disproportionally high number of functional mutations, but showing no evidence of pseudogenization. Several pairs of paralogs are asymmetric in the rate of synonymous substitutions although this result is not significant on the genome scale.

## Methods

### Identification of gene families

Validated RefSeq mRNA collection [40,41] available at the UCSC Human Genome Browser [42,43] was used as human genes markers. Overlapping mRNAs were linked together. Repeats were removed using RepeatMasker [44,45]. Then masked sequences were compared pairwise by using discontiguous megablast [46,47] with the following parameters: all filters set up (including Dust, human repeats, vector screen, and low-complexity regions), X-dropoff value set to 150, discontiguous word template length set to 18, discontiguous templates were both coding and non-coding, and word size set to 11. Gene pairs with at least 75% identity at more than 20% of their average length were linked. Single linkage clustering was used to form preliminary gene groups.

Finally, ClustalW [48,49] was used to align protein-coding regions of all family members. The alignments were manually curated. After gap removal all families with less than 25% or more than 98% identical nucleotide positions in the multiple alignment were filtered out. Single genes diverging from other family members at more than 80% nucleotide positions on average also were filtered out. We used only gene families containing three or more genes. This procedure, aimed at identifying very recent paralogs, treats single-exon genes and genes with multiple-exons differently. For genes with introns it usually requires some intron similarity and thus guarantees that duplications are recent, whereas the single-exon gene families may have diverged earlier. To take the influence of single-exon genes into account we validated all general observations separately on single-exon and multiple-exon gene subsets.

We manually curated gene families combining single-exon genes and genes with multiple-exons. All genes suspicious for pseudogenization (3 cases) were removed from the study. In one case the removal of the processed pseudogene led to the exclusion of the whole gene family, as it ended up having only two genes.

### dN/dS calculations

The values of dN and dS were calculated using the codeml program from the PAML program package [50,51]. The default parameters, except "runmode = -2 (pairwise)" were used. Output pairwise values of dS were used to determine the sister genes and the cousins, and pairwise dS and dN values were used to calculate individual dS and dN values using the method described in "Estimation of individual dN and dS rates" (Results).

We used the average pairwise dN and dS values of all gene family members as dN and dS values of gene families.

### Study of asymmetry

If gene A is a sister of gene B and gene B is a sister of gene A, the genes are considered bidirectional best paralogs. We then searched for the closest cousin for the pair of genes and used it as an outgroup. If genes A and B are bidirectional best paralogs, gene C is the closest cousin to the pair of genes A and B if the dS value between genes A and C and dS value between genes B and C is larger than the dS value between genes A and B, and if the sum of dS values from cousin C to both genes from the pair is the smallest among any all possible cousins. The amino acid sequences of the encoded proteins were compared. If all three genes differed at a position, this position was not considered in the analysis. At all other divergent positions the outgroup was used to identify which gene from the pair acquired a substitution since the duplication event. The number of such amino acid substitutions in proteins encoded by each gene of a pair was calculated.

The accumulation of neutral mutations in a DNA segment is usually assumed to follow a Poisson process [52] and thus the distribution of the number of substitutions in each paralog (X_1 _and X_2_, for a paralogous pair) is a Poisson one. The hypothesis of the symmetry of paralog evolution implies the coincidence of the distribution coefficients. In this case if the substitutions in paralogs are independent, the conditional distribution of the substitution number in one gene of a pair assuming the fixed total number of substitutions, i.e. (X_1 _| Y = X_1 _+ X_2_), is binomial with p = q = 1/2 [53].

We checked the hypothesis of the symmetric binomial distribution (p = q = 0.5) of substitutions between the genes of each pair. If the statistical significance of the hypothesis (estimated by P-value) was less than 0.05, the gene pair was considered asymmetrically evolving. To estimate the statistical significance of the observed number of asymmetrically evolving gene pairs we checked the hypothesis of the binomial distribution (p = 0.05) of observed outcomes.

### Orthology relationships

Translates of all genes from the studied triplets were compared to translates of the mouse CCDS protein database from the Human Genome Browser using BlastP (word size = 2; E-value cut-off = 10). The best hit from mouse was added to the sequence list. A Neighbor-Joining tree was calculated using ClustalW (distance correction = true, ignore gaps = true). We then checked whether any orthologs were closer to a paralogous gene pair than their cousin.

### Database comparison

We used GOstat [25,54] to analyze over- and underrepresentation of gene functions between all genes in our dataset and the human transcriptome and between asymmetrically evolving genes and all other genes of our dataset. We used the UniGene [27] dataset to obtain EST data for the studied genes.

### PolyPhen analysis

All amino acid substitutions in proteins encoded by paralogous gene pairs used for the asymmetry study were classified into "damaging" and "neutral" by a new version of PolyPhen, a computational tool for prediction of possible functional and structural impact of amino acid substitutions in proteins [55]. The chi-square analysis was used to search for differences in the distribution of substitutions.

## Competing interests

The authors declare that they have no competing interests.

## Authors' contributions

AYP and IIA designed the study, performed the analysis and wrote the manuscript. MSG participated in planning the research and contributed to the manuscript. VER performed the PolyPhen analysis and contributed to the manuscript. All authors read and approved the final manuscript.

## Reviewers' comments

### Reviewer #1: Dr. Igor Rogozin

The authors analyzed recent gene duplications of human genes. They found that ~20% of genes in paralogous gene families are evolving asymmetrically: one of the proteins encoded by two closest paralogs accumulates amino acid substitutions significantly faster than its partner. Another interesting observation is that the increase of negative selection pressure over time after a duplication event seems to be a global trend in the evolution of human paralogous gene families.

#### Comments

1) The authors measured correlation between X/Y and Y (X = dN, Y = dS). These two variables are related by the definition. By another definition correlation determines whether values of one variable are related to another. Furthermore, we know that X and Y are weakly positively correlated. Sure, you can measure the correlation between related variables, this analysis can produce a negative correlation between X/Y and Y. This indicates that there is a correlation between related variables X/Y and Y (theoretically there may be no linear correlation between related

variables). However this correlation does not immediately suggest that "The increase of negative selection pressure over time after a duplication event seems to be a global trend in the evolution of human paralogous gene families". Such conclusions can be safely made if X/Y and Y would be independent random variables but they are not. I think that the interpretation requires major caution.

2) The authors used ClustalW [41,42] to align protein-coding regions of all family members. In general it is better to align amino acid sequences first. Nucleotide sequences are aligned to correspond to the amino acid sequence alignments (maintain reading frame).

3) Potential problems resulted from aligning of nucleotide protein-coding sequences can be avoided by manual curation. This was done by the authors. Still I am suspicious about obvious outliers with dN/dS >2 (Figure 2). This might be a result of frameshifts in alignments.

### Authors response

It is indeed true that dN and dS are somewhat correlated. A perfect correlation between dN and dS would result in a linear function dN = k*dS if negative selection (dN/dS) did not increase over time (dS). Under these circumstances dN/dS would be described by a horizontal line dN/dS = k. This is the null hypothesis. Instead we observe a function for dN/dS from dS that is incompatible with the null hypothesis. Our linear regression shows that dN/dS = k1*dS + k (with a negative k1 and positive k) is a better approximation to the curve than dN/dS = k in the 95% confidence interval. This means that not only dN increases with dS (which is obvious), but the rate of dN increase changes with dS which is basically the statement we make.

Manual curation of alignments was used to ensure that frameshifts are not present. We removed several pseudogenes from our study. Some of these removed pseudogenes contained frameshifts. The remaining genes did not.

The alignment algorithm for coding nucleotide sequences using amino acid sequences first, as suggested by the reviewer is a good alternative to certain steps of our manual curation process. Although we did not use the proposed sequence of actions, we did use amino acid sequences subsequently when curating the nucleotide alignments.

### Reviewer #2: Dr. Fyodor Kondrashov

This paper presents three main results:

1) dN/dS declines with dS.

2) 18% of paralogous gene pairs evolve asymmetrically when the relative rates are tested with a more distant paralog is used as an outgroup.

3) The fraction of predicted deleterious substitutions is much higher in the fast evolving member of a recent duplicate pair.

Generally, I found the paper boring at times, especially with the introduction repeating the discourse that has been tackled with over the last decade at least. More so, it seemed to me that while the paper analyzed interesting results it did not leave me as a reader feeling completely satisfied. I would suggest rewriting the paper either doing a more complete literature review, or stating the tested hypotheses and the reasons why they are tested more succinctly.

My major concern deals with the first result being a possible artifact of taking fractions of very small values. It would be worthwhile for the authors to invest some time showing that dN/dS values < 0.05 are not systematically overestimated due to the nature of discrete counting of the number of substitutions.

On a more minor level, I think it would be worthwhile for the authors to at least mention the issue of gene conversion, which is especially important here because the outgroup may also be subject to converting with the sister species. If this occurs across few exons the authors may not be measuring dN and dS values correctly across the entire gene. Also, because gene families are selected this automatically pre-selects genes with non-random gene functions, that is more non-random that all possible gene duplications, which may have an effect on the proportion of observed asymmetrically evolving gene copies.

Finally, I believe that the strongest and the most novel result of the paper is the large skew of predicted deleterious substitutions in the gene copies and that it deserved greater attention.

Another minor point: the use of the first two citations is strange, I am sure that more appropriate citations can be found for the theoretical-methodological background they are cited for initially.

### Authors response

We are grateful to the reviewer for pointing out that we did not exclude the effect of discrete counting on extreme values of dN/dS. We have performed an additional test and added the following text:

"As an additional control we restricted the sample to genes that had at least 100 substitutions, when compared to their sister gene. This was done to ensure that the observed trends are not caused by discrete substitution counts. The linear regression coefficient for dN/dS from dS in this subsample of 232 genes remained negative and was equal to -1.31; 95% confidence interval (-1.94; -0.69)."

We acknowledge that gene conversion might have happened in some genes from our dataset and that in certain regions of genes we might be measuring dN and dS from the last gene conversion effect instead of measuring dN and dS from the last duplication event. These cases are hard to identify. However, gene conversion by itself could not result in the observed trend of dN/dS decrease over time.

To artificially produce the observed trend of dN/dS decrease with increasing dS any unaccounted effect (such as gene conversion) is required to enrich the dataset with points that have either larger dN/dS and smaller dS, or smaller dN/dS and larger dS than they would otherwise. Assume that dN/dS does not increase with decreasing dS, but does not change or decreases instead. Then a gene that has undergone conversion would consist of an older part (unchanged dN/dS and dS) and a younger part (smaller dS and, by the above assumption, unchanged or smaller dN/dS). In these circumstances the conversion would enrich the dataset with points that have smaller dS and smaller dN/dS or unchanged dN/dS, not larger dN/dS as required. Thus, gene conversion could not artificially produce the observed trend if it did not exist all along.

The reviewer is correct to notice that our dataset is biased towards genes with certain functions. This is described in detail in the subsection "Individual Kn and Ks values of paralogous genes". Of course, the proportions of gene pairs that evolve asymmetrically might depend on gene properties. Unfortunately, maybe due to a small statistical power, we cannot identify traits that are associated with asymmetric evolution. Hence we state that "according to a GOstat comparison, genes from asymmetrically evolving gene pairs are not significantly different (P < 0.01) from other genes of the initial dataset in terms of protein function".

We agree with the reviewer that "the most novel result of the paper is the large skew of predicted deleterious substitutions in the gene copies". This is mentioned in our abstract, results and discussion. We tried to highlight this finding a bit more in the final version of the article.

Following the reviewer's suggestion we also rewritten and restructured the "background" section of the manuscript and added a number of appropriate references.

### Reviewer #3: Dr. Sergei Maslov

This reviewer provided no comments for publication.
